# Methylation panel is a diagnostic biomarker for Barrett’s oesophagus in endoscopic biopsies and non-endoscopic cytology specimens

**DOI:** 10.1136/gutjnl-2017-314026

**Published:** 2017-10-30

**Authors:** Hamza Chettouh, Oliver Mowforth, Núria Galeano-Dalmau, Navya Bezawada, Caryn Ross-Innes, Shona MacRae, Irene Debiram-Beecham, Maria O’Donovan, Rebecca C Fitzgerald

**Affiliations:** 1 MRC Cancer Unit, Hutchison/MRC Research Centre, University of Cambridge, Cambridge, UK; 2 Department of Histopathology, Addenbrooke’s Hospital, Cambridge, UK

**Keywords:** hypermethylation, non-endoscopic cell sampling, biomarker, barrett’s, reflux, cytosponge

## Abstract

**Objective:**

Barrett’s oesophagus is a premalignant condition that occurs in the context of gastro-oesophageal reflux. However, most Barrett’s cases are undiagnosed because of reliance on endoscopy. We have developed a non-endoscopic tool: the Cytosponge, which when combined with trefoil factor 3 immunohistochemistry, can diagnose Barrett’s oesophagus. We investigated whether a quantitative methylation test that is not reliant on histopathological analysis could be used to diagnose Barrett’s oesophagus.

**Design:**

Differentially methylated genes between Barrett’s and normal squamous oesophageal biopsies were identified from whole methylome data and confirmed using MethyLight PCR in biopsy samples of squamous oesophagus, gastric cardia and Barrett’s oesophagus. Selected genes were then tested on Cytosponge BEST2 trial samples comprising a pilot cohort (n=20 cases, n=10 controls) and a validation cohort (n=149 cases, n=129 controls).

**Results:**

Eighteen genes were differentially methylated in patients with Barrett’soesophagus compared with squamous controls. Hypermethylation of TFPI2, TWIST1, ZNF345 and ZNF569 was confirmed in Barrett’s biopsies compared with biopsies from squamous oesophagus and gastric cardia (p<0.05). When tested in Cytosponge samples, these four genes were hypermethylated in patients with Barrett’s oesophagus compared with patients with reflux symptoms (p<0.001). The optimum biomarker to diagnose Barrett’s oesophagus was TFPI2 with a sensitivity and specificity of 82.2% and 95.7%, respectively.

**Conclusion:**

TFPI2, TWIST1, ZNF345 and ZNF569 methylation have promise as diagnostic biomarkers for Barrett’s oesophagus when used in combination with a simple and cost effective non-endoscopic cell collection device.

**Significance of this study**

**What is already known about this subject?**

- Methylation changes occur early in Barrett’s carcinogenesis.
- Hypermethylation of TFPI2 and TWIST1 genes occurs commonly in cancer including oesophageal adenocarcinoma.
- Barrett’s oesophagus is underdiagnosed due to reliance on endoscopy. Non-endoscopic cell collection using the Cytosponge has potential as a screening tool for Barrett’s oesophagus when coupled with a biomarker.

**What are the new findings?**

- We have identified 18 genes that are differentially methylated in Barrett’s oesophagus compared with normal squamous oesophagus, some of which are potential diagnostic biomarkers.
- Four methylation markers (TFPI2, TRIST1, ZNF345 and ZNF569) can detect Barrett’s oesophagus when applied to Cytosponge samples.

**How might it impact on clinical practice in the foreseeable future?**

- This quantitative methylation biomarker has promise as a diagnostic tool for Barrett’s oesophagus without the need for endoscopy.

## Introduction

Barrett’s oesophagus is a metaplasia of the normal stratified squamous epithelium of the distal oesophagus to a columnar epithelium, which generally occurs in individuals with chronic exposure to gastric acid and bile reflux. This metaplasia has been shown to be a risk factor for oesophageal adenocarcinoma (OAC) which, unless detected early, has an overall mortality above 80% at 5 years.^1 2^ Moreover, in the western world, the incidence of OAC has rapidly increased in recent decades, such that it now constitutes more than 50% of cases of oesophageal cancer,^3–6^ making diagnosis of Barrett’s oesophagus all the more pertinent.

The clinical relevance of diagnosing Barrett’s oesophagus lies in the opportunity for early detection of dysplasia and superficial carcinoma, for which curative non-surgical treatment options, such as radiofrequency ablation and endoscopic mucosal resection, have become widely available in the past 10 years.^7^

Currently, the diagnosis of Barrett’s oesophagus relies on endoscopy in patients who are referred with symptoms. In the UK and the USA, only patients with chronic GORD and multiple risk factors (at least three of: age 50 years or older, white race, male sex and obesity) are currently recommended for screening endoscopy by best practice guidelines,^8^ otherwise it is often an incidental finding and the majority of cases are undiagnosed.^9^ A fundamental argument against screening arises from the low conversion rate of Barrett’s oesophagus to OAC, with one population-based pathology study estimating the absolute annual risk of OAC associated with Barrett’s oesophagus may be as low as 0.12%,^10^ although a meta-analysis suggested a conversion rate of 0.3%.^11^ Moreover, endoscopic diagnosis is invasive and expensive and requires expert training, making it unfeasible as a population screening tool.

Alternative diagnostic techniques such as office-based transnasal endoscopy and string-capsule video endoscopy^12 13^ have been considered, although the problems of expense and specialist training remain. Consequently, non-endoscopic screening tools have been developed in attempts to overcome these cost and training issues. One such tool is the Cytosponge, a non-endoscopic cell collection device that comprises a polyurethane sponge contained within a gelatine-based capsule, which is attached to a thin string. The patient swallows the capsule with some water while holding on to the other end of the string that is attached to a retainer. The capsule dissolves over a few minutes in the proximal stomach; the sponge is then retrieved 5 min later, scraping a sample of more than 500 000 cells from the lining of the oesophagus in the process, which can then be analysed for biomarkers to maximise the accuracy and objectivity of the diagnosis.^14 15^

The Cytosponge has been found to be feasible in the primary care setting and acceptable to patients,^14^ suggesting that this inexpensive, minimally invasive tool may be appropriate as a triaging test for general practitioners. Furthermore, initial health economic analyses have suggested that the cost of this test is favourable when coupled with endoscopic therapy for dysplasia.^16^ The biomarker that has been tested extensively to date is an immunohistochemical biomarker, trefoil factor 3 (TFF3), which is a mucin-associated peptide that has been shown to distinguish Barrett’s oesophagus cells from normal squamous epithelium and gastric cardia with a sensitivity of 79.9% and specificity of 92.4% in a multicentre case–control study.^17^ The sensitivity increases with the length of the segment to 87.2% in segments of circumferential Barrett’s oesophagus ≥3 cm and is preserved in dysplasia.^15^ While the accuracy is favourable compared with other screening tests,^18–20^ ideally accuracy would be improved further and although an advantage of the TFF3 result is its binary nature, it would be ideal if analysis did not require paraffin embedding and a pathological examination. Therefore, we sought to explore the use of DNA methylation in view of promising data in other contexts.^21^

DNA methylation is a phenomenon whereby a methyl group is added to the carbon-5 position of cytosine at CpG sites, which consist of a cytosine located adjacent to guanine. Crucially, this epigenetic change is thought to influence gene expression and hence may be a contributor to oncogenesis. CpG islands, areas of DNA with high GC content, have been found to be hypermethylated in cancer cells.^21^ Aberrant DNA methylation has been identified in a number of tumour suppressor, DNA repair and adhesion molecule genes in Barrett’s cells, such as AKAP12,^22^ APC^23 24^ and GPX3.^25 26^ Such studies have elucidated that promoter hypermethylation is commonly an early but progressive aberration in the Barrett’s oesophagus–OAC sequence,^27^ suggesting that differential methylation of such genes may prove useful as biomarkers in detecting Barrett’s oesophagus and may also have value in risk stratification.

The overall objective of this study was therefore to discover, test and validate new methylation biomarkers and to test their performance when applied to a non-endoscopic cell-collecting device—the Cytosponge.

## Methods

### Identification of differentially methylated genes from methylation array

We analysed the beta values from a relevant, publicly available dataset generated from an Illumina 27k array analysis.^28^ We first filtered the probes and kept only those that covered gene CpG islands. A non-paired Wilcoxon test was applied to determine significant differentially methylated genes between normal squamous biopsies and Barrett’s samples with p<0.05.

### Cohorts design and ethics

Both biopsies and Cytosponge tissues were selected from the BEST2 trial.^17^ Ethics approval for this study had been obtained from the East of England – Cambridge Central Research Ethics Committee (No: 10/H0308/71) and registered in the UK Clinical Research Network Study Portfolio (9461). Individual written informed consent was obtained for each patient including permission to perform additional biomarker research. The BEST2 study is registered with ISRCTN, number 12730505.

### Cytosponge processing

The stored paraffin blocks from non-dysplastic Barrett’s cases and controls (without Barrett’s oesophagus) from the BEST2 trial Cytosponges were used as well as standard FFPE blocks from endoscopic biopsies. For Cytosponge processing, the entire cell pellet is embedded in a single paraffin block as previously described.^17^ Cases were selected from the database to include all non-dysplastic BE cases and controls with adequate material remaining in the block for a methylation assay. No consideration was given to the proportion of columnar cells. Genomic DNA was extracted from 8×10 µm sections using Deparaffinisation Buffer (Qiagen, Manchester, UK) and the QIAamp FFPE DNA Tissue Kit (Qiagen). The protocol was followed as described by the manufacturer with the exception that samples were incubated at 56°C overnight instead of the described 1 hour, and 10 µL of extra Proteinase K was added to the samples roughly halfway through the 24-hour incubation.

### Bisulfite modification and MethyLight PCR

Extracted DNAs were bisulfite converted by following EZ DNA-Methylation Gold kit instructions (Zymo Research, Irvine, California, USA). For MethyLight PCR, a TaqMan approach was carried out as described by Eads and collaborators.^29^ Each PCR reaction included 1× of LightCycler 480 Probes Master Mix (Roche, Welwyn Garden City, UK), 100 µM final concentration of probe, 10 µM final concentration of each forward and reverse primer, DNAse free water up to 8 µL final volume and 2 µL of bisulfate-converted DNA. Both probes and primers were generated using BEACON software, and the corresponding sequences are shown in online supplementary table 1. Primers and probes were first tested on a Universally Methylated DNA (UM-DNA) (Millipore, Watford, UK) for amplifying methylated DNA only after bisulfate modification. Standard curves were generated for each gene of interest using a bisulfate converted UM-DNA, and a calibrator (corresponds to the 1:100 dilution of UM-DNA) was used in all subsequent experiments to allow absolute quantification. Each sample was analysed in triplicate, and the methylation level was calculated as follows: methylation=(A/B)/(C/D), wherein A=value of methylation of gene of interest in each sample; B=value of methylation of the gene of interest in the calibrator; C=level of amplification of β actin in each sample; and D=level of amplification of β actin in the calibrator.

10.1136/gutjnl-2017-314026.supp2 Supplementary file 2

### Statistical analysis

To achieve a power of 0.95 (0.05 error) for a large effect size (comparable with area under the curve (AUC) of 0.8), we needed a minimum of 52 samples, and we therefore used all the non-dysplastic and control samples from the BEST2 trial with adequate material for analysis. We used the non-paired Wilcoxon test to compare the methylation level between Barrett’s oesophagus and normal samples. For demographic statistics, the χ^2^ test was used to compare categorical variables, and the Pearson correlation coefficient was calculated between the percentage of gene methylation and demographic factors. The receiver operating characteristic (ROC) analysis was performed using the pROC package to identify sensitivity, specificity and the AUC. The ROC analysis was also used to determine the methylation cut-off for each gene in the discovery cohort to be applied on the validation cohort.

## Results

### Differential gene methylation in Barrett’s biopsies compared with control tissues

In order to identify differentially methylated genes in Barrett’s oesophagus compared with normal squamous tissue, we reanalysed a dataset from our laboratory that had originally been generated to assess methylation changes in malignant progression.^28^ This dataset assessed the methylome of 22 of Barrett’s samples (biopsies), 24 OAC samples and also included two squamous oesophageal biopsies as internal control samples. We found 18 hypermethylated genes in Barrett’s oesophagus compared with normal tissue with a difference in methylation of between 30% and 75% of methylation and with a p<0.05 (online supplementary figure 1A). Using unsupervised hierarchical clustering, the methylation level of these candidates clustered all Barrett’s samples together indicating a potential diagnostic value of these candidates (online supplementary figure 1B). Four of these genes (APC, EYA4, RBP1 and SFRP4) have previously been reported to be hypermethylated in Barrett’s oesophagus lending support to our candidate gene list.^23 30–33^ It is also interesting that some other genes that had been reported to be differentially methylated previously (CDX2, B3GAT2 and vimentin) were found to be more highly methylated in Barrett’s oesophagus compared with normal squamous biopsies that did not reach statistical significance (<0.05) and so were not taken forward (online supplementary figure 2).

10.1136/gutjnl-2017-314026.supp1 Supplementary file 1

**Figure 1:**
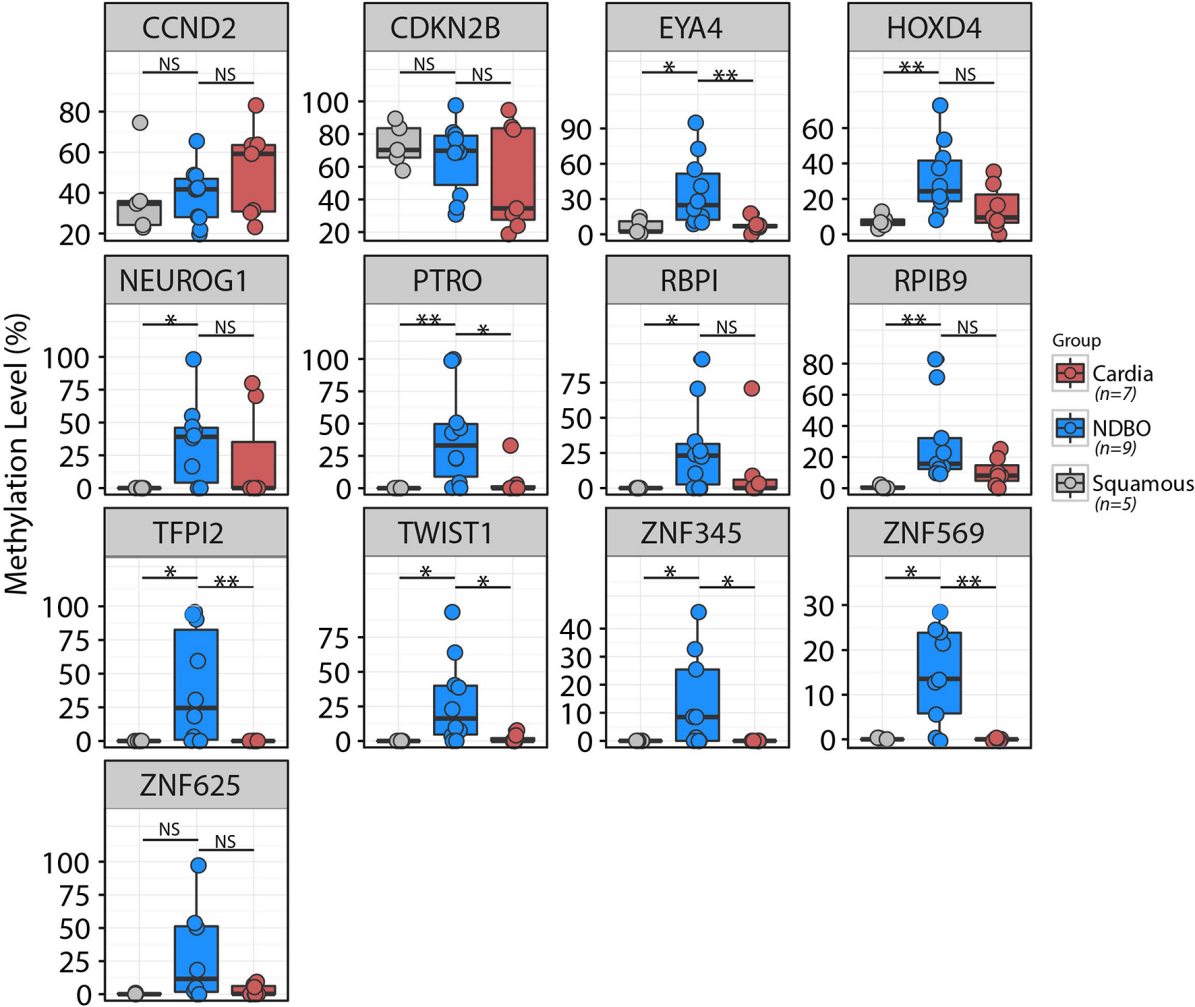
Testing methylation level of gene candidates in a biopsy cohort. The methylation level of 13 genes was assessed by MethyLight PCR in five squamous, seven cardia and nine Barrett’s biopsies. Ten out of 13 genes were significantly hypermethylated in Barrett’s biopsies compared with normal squamous tissue (*p<0.05; **p>0.01). Only TFPI2, TWIST1, ZNF345 and ZNF569 were specifically hypermethylated in Barrett’s samples and not in squamous tissue nor cardia biopsies.

In order to confirm whether the candidates identified from the methylation array were indeed differentially methylated in Barrett’s oesophagus compared with normal squamous tissues, we first performed MethyLight PCR in an independent biopsy cohort comprising nine confirmed Barrett’s samples and five normal squamous biopsies. Thirteen out of 18 candidates passed primer testing. CCND2, CDKN2B and ZNF625 methylations were not significantly different between Barrett’s oesophagus and squamous tissues. The 10 other genes were significantly hyper-methylated in Barrett’s oesophagus compared with normal squamous tissue (HOXD4, PTRO and RPIB9 with p<0.01 and the rest of the genes with p<0.05) (figure 1). Since the Cytosponge collects cells from the gastric cardia as well as from oesophageal tissues, it is important that any biomarker can also discriminate Barrett’s from cardia cells. Therefore, we also assessed the methylation level of these 13 candidates in gastric cardia tissues. This identified four genes, TFPI2, TWIST1, ZNF345 and ZNF569, that were hypermethylated in Barrett’s oesophagus but not in either of the control tissues (p<0.01) (figure 1).

### Testing the four-gene methylation panel in a Cytosponge case: control cohort

To evaluate whether the methylation level of these four genes could act as a diagnostic biomarker for Barrett’s oesophagus using the Cytosponge, we used two cohorts: a pilot cohort (n=10 controls; n=20 Barrett’s oesophagus) and a validation cohort (n=129 controls, n=149 Barrett’s oesophagus) from the Barrett’s Oesophagus Screening trial (BEST2). The BEST2 trial enrolled patients with reflux symptoms and no endoscopic evidence of Barrett’s oesophagus (controls) as well as patients with known Barrett’s oesophagus (cases).^17^ The demographic and clinical characteristics were as expected for the controls versus Barrett’s cases, and these characteristics were maintained when randomly assigned to the pilot and validation cohorts (table 1).

**Table 1:** Patient demographics of pilot and validation Cytosponge cohorts

	Controls			Cases			Pilot	Validation
Characteristic	Pilot cohort	Validation cohort	p Value	Pilot cohort	Validation cohort	p Value	(Controls vs cases)	(Controls vs cases)
	(n=10)	(n=129)		(n=20)	(n=149)		p Value	p Value
Age (years, median (IQR))	48.0 (39.0–61.0)	55.0 (43.0–63.0)	0.2985	66.0 (58.0–75.0)	65.0 (56.0–72.0)	0.4771	0.003	<0.0001
Gender (n (%))								
Female	7 (70.0)	72 (55.8)	0.5884	6 (30.0)	27 (18.1)	0.3381	0.09	<0.0001
Male	3 (30.0)	57 (44.2)		14 (70.0)	122 (81.9)			
Ethnicity (n (%))			0.4654			0.6611	0.28	0.2149
White British	9 (90.0)	113 (87.6)		19 (95)	139 (93.3)			
Other white background	0	11 (8.5)		1 (5)	5 (3.4)			
Asian	1 (10.0)	2 (1.6)		0	5 (3.4)			
African	0	2 (1.6)		0	0			
Other	0	1 (0.8)		0	0			
Body mass index (kg/m^2^, median (IQR))	27.7 (24.0–29.2)	27.4 (24.5–30.4)	0.6531	28.3 (25.2–29.9)	28.4 (25.7–30.8)	0.6143	0.67	0.106
Waist:hip (median (IQR))	0.87 (0.81–0.88)	0.87 (0.82–0.94)	0.5227	0.92 (0.90–0.95)	0.95 (0.92–0.99)	0.06754	0.002	<0.0001
Hiatus hernia	3 (30%)	46 (35.7%)	0.9722	17 (85%)	118 (79.2%)	0.7557	0.009	<0.0001
Barrett’s length								
C (cm)	–	–		3.0 (0.4–6.3)	2.0 (1.0–5.0)	0.8188		
M (cm)	–	–		5.0 (3.0–7.0)	4.0 (3.0–7.0)	0.7306		

**Figure 2:**
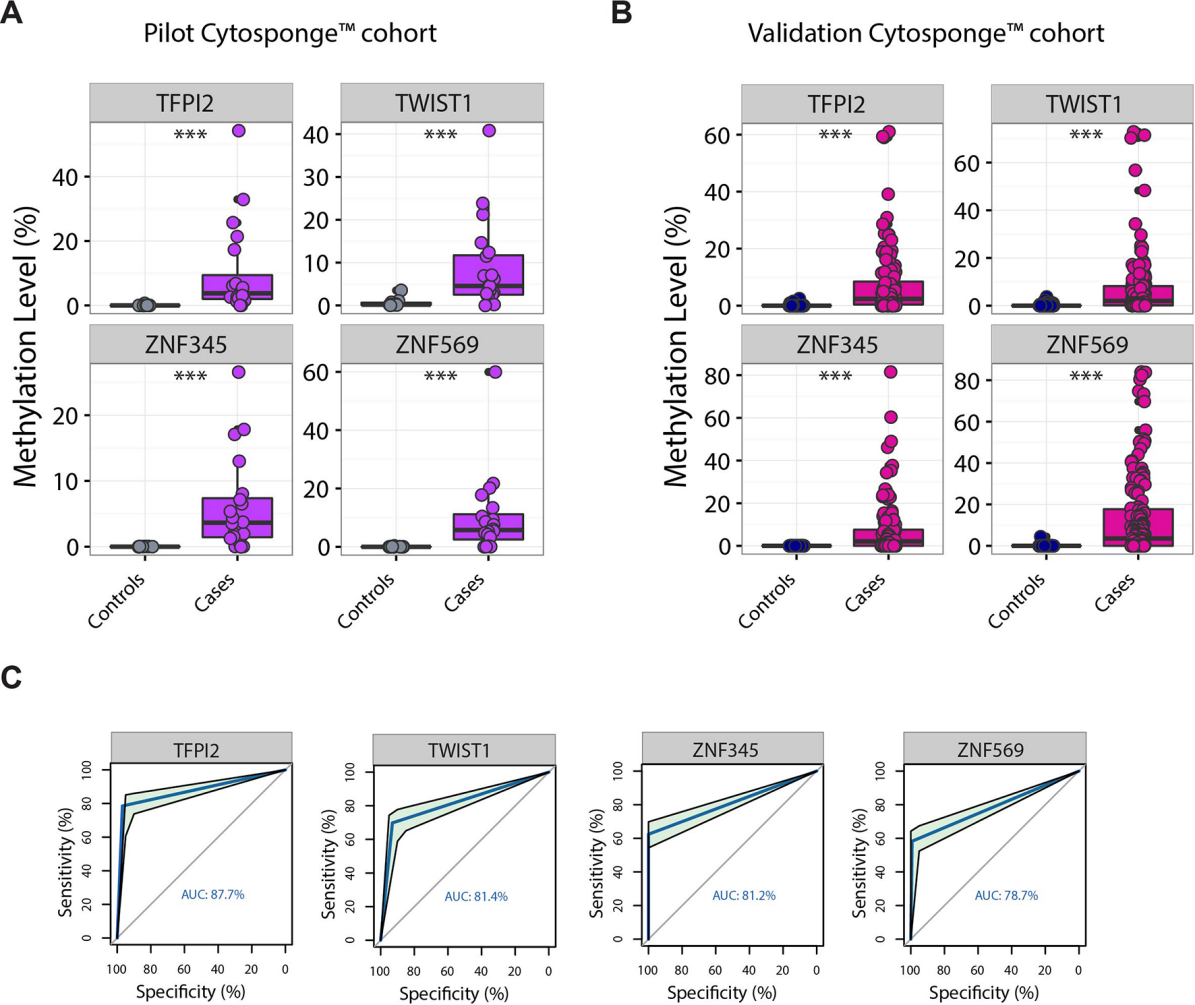
Test and validation of the four gene candidates in a Cytosponge cohort. TFPI2, TWIST1, ZNF345 and ZNF569 were significantly hypermethylated in a pilot Cytosponge cohort (A) from patients with Barrett’s oesophagus compared with those from control patients (***p<0.0001). The pilot cohort included 20 Barrett’s oesophagus and 10 controls. Results were confirmed in a larger Cytosponge cohort from the BEST2 study (B) (***p<0.0001). The validation cohort had 149 Barrett’s oesophagus and 129 controls. (C) ROC curves for selected methylated genes in the validation Cytosponge cohort using thresholds identified in the pilot cohort. ROC, receiver operating characteristic.

In the pilot cohort, all four genes were hypermethylated in the Cytosponge samples from patients diagnosed with Barrett’s oesophagus compared with the controls (figure 2A, p<0.001). We therefore proceeded to a validation cohort and again found that all four genes were significantly hypermethylated in Barrett’s oesophagus samples compared with controls (figure 2B, p<0.001). An ROC analysis was performed in order to estimate the specificity and sensitivity for each marker separately (figure 2C and table 2). ZNF569 had the lowest AUC (0.79) with a specificity and sensitivity of 99.2% and 59.1%, respectively (95% CI 75.1% to 83.2%), whereas TFPI2 was the best candidate with an AUC of 0.88. Since specificity is critical for a diagnostic biomarker in a disease of low prevalence in the population, the optimal specificity and sensitivity for TFPI2 were 96.9% and 78.5%, respectively (95% CI 84.1% to 91.3%).

**Table 2:** ROC analysis results on the validation Cytosponge cohort

	Specificity (%)	Sensitivity (%)	AUC (%)	**95%** CI	Threshold (%)
TFPI2	96.9	78.52	87.7	84.08 to 91.34	0.94
TWIST1	93.02	69.8	81.4	77.1 to 85.72	1.47
ZNF345	100	62.42	81.2	77.31 to 85.11	0.29
ZNF569	99.22	59.06	78.7	75.11 to 83.18	0.03

AUC, area under the curve; ROC, receiver operating characteristic.

### Relationship of methylation levels to clinical variables

**Figure 3:**
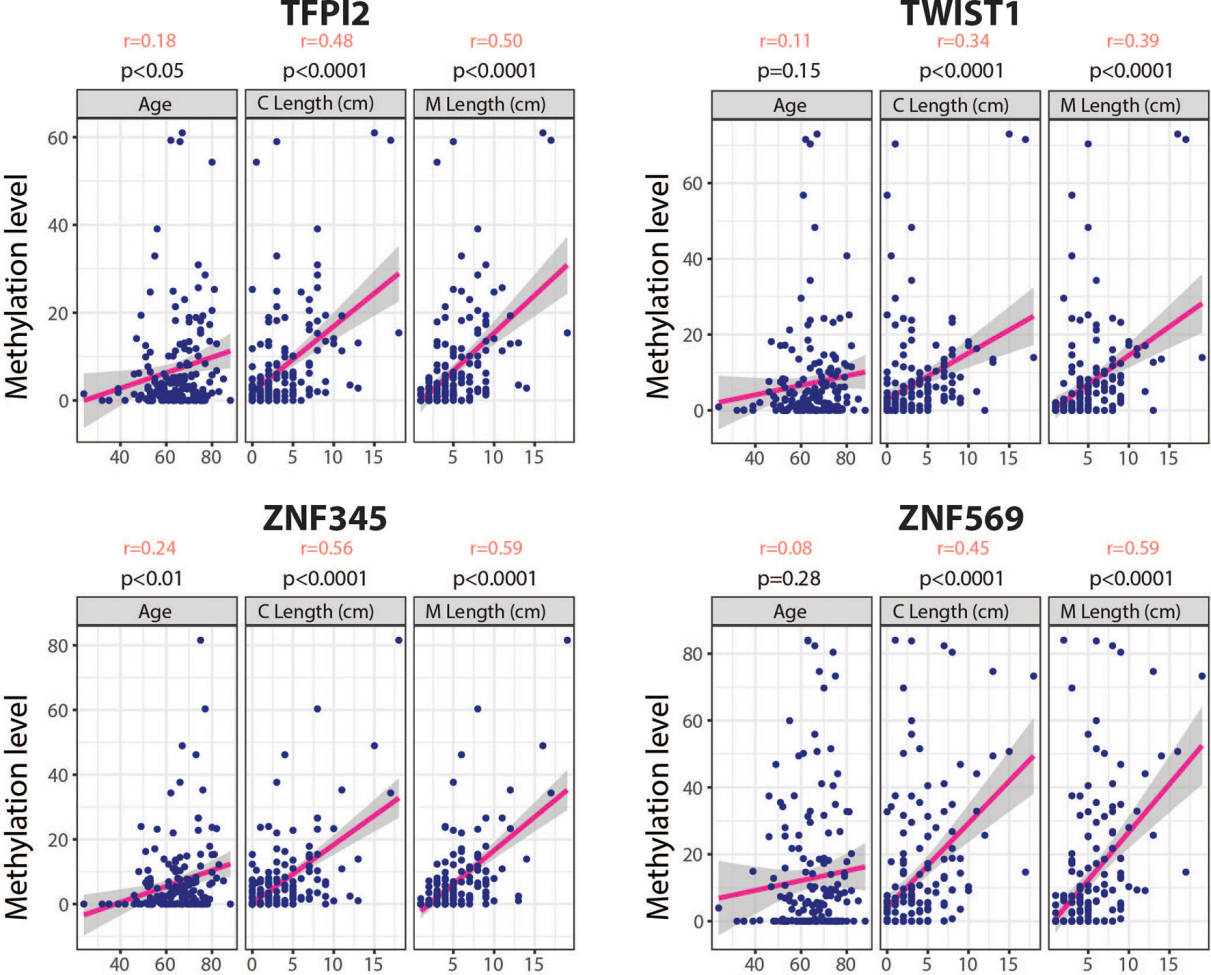
Correlation of gene methylation status and patient age and Barrett’s segment length. Dotplot showing correlation between the 4 methylation biomarkers (TFPI2, TWIST1, ZNF345, ZNF569) and age, circumferential (C) and maximal (M) Barrett’s lengths. The r- value represents the Pearson correlation coefficient for each variable.

The relationship between the methylation levels of these genes and demographic characteristics in the cases are presented in table 3. We did not observe any correlation with body mass index (BMI) and waist-to-hip ratio. In contrast, there was a significant correlation between methylation levels in the gene signature panel and patient age ([p<0.05, r=0.18]. This was significant when considering genes individually for ZNF345 and TFP12, [(p<0.01, r=0.24] and [(p<0.01, r=0.18] respectively), but not for TWIST1 nor ZNF569. A significant and stronger correlation was also observed for all genes in relation to the circumferential and maximal lengths of the Barrett’s segment (C & M) (table 3 and figure 3). Moreover, we found that there is a linear relationship between sensitivity of the biomarkers and the length of the Barrett’s segment (r= (0.94–0.99) and r= (0.99–1.00) for C and M respectively), which are both known risk factors for progression to dysplasia and cancer (online supplementary figure 3).

**Table 3:** Correlation of identified genes with patient characteristics in cases

Gene signature	r Pearson	p	Rho spearman	p
Age	0.18	0.019	0.18	0.016
BMI	0.03	0.6555	0.03	0.6865
Waist:hip ratio	0.05	0.5104	0.08	0.2972
C	0.58	<0.0001	0.54	<0.0001
M	0.60	<0.0001	0.60	<0.0001

TFPI2	r Pearson	p	Rho spearman	p
Age	0.18	0.021	0.22	0.004
BMI	0.02	0.7421	0.05	0.5091
Waist:hip ratio	0.05	0.4819	0.06	0.4342
C	0.48	<0.0001	0.51	<0.0001
M	0.50	<0.0001	0.57	<0.0001

TWIST1	r Pearson	p	Rho spearman	p
Age	0.11	0.1473	0.19	0.011
BMI	−0.10	0.1814	−0.002	0.9793
Waist:hip ratio	0.06	0.4369	0.08	0.3327
C	0.34	<0.0001	0.44	<0.0001
M	0.39	<0.0001	0.47	<0.0001

ZNF345	r Pearson	p	Rho spearman	p
Age	0.24	0.001	0.25	0.001
BMI	−0.02	0.8297	0.05	0.5483
Waist:hip ratio	0.06	0.4418	0.14	0.0749
C	0.56	<0.0001	0.48	<0.0001
M	0.59	<0.0001	0.57	<0.0001

ZNF569	r Pearson	p	Rho spearman	p
Age	0.08	0.2826	0.10	0.1869
BMI	0.06	0.4577	0.06	0.4631
Waist:hip ratio	0.03	0.7327	0.04	0.5744
C	0.45	<0.0001	0.48	<0.0001
M	0.59	<0.0001	0.51	<0.0001

BMI, body mass index; C, circumferential; M, maximal extent of Barrett’s.

## Discussion

In this study, we have demonstrated that 10 genes are hypermethylated in Barrett’s biopsies compared with normal squamous tissue and these included six novel targets as well as confirming some genes previously reported in the literature.^30–35^ Of these, TFPI2, TWIST1, ZNF345 and ZNF569 genes were the most differentially methylated in Barrett’s oesophagus compared with neighbouring normal squamous oesophagus and gastric cardia tissues that are important for application to the Cytosponge, which samples cells from these adjacent sites.

TFPI2 is a tumour suppressor gene that is known to be hypermethylated in many tissue types including the gastrointestinal tract^36–38^ and is one of the top 20 genes for detecting early stage oral squamous cell carcinoma.^39^ Its tumour suppressor properties are due to its inhibitory effects on protease activity, thus protecting the tumour cell matrix from degradation and counteracting malignant cell invasion and metastasis. Methylated TFPI2 has been identified as a potential cancer biomarker with particular relevance to the early detection of cancer. For example, in stool samples, it has been shown to distinguish colorectal cancer and colorectal adenomas from healthy individuals, with a sensitivity of 76%–89% and a specificity of 79%–93%.^40^ In squamous oesophagus carcinogenesis, methylation of the TFP12 promoter was shown to occur early at dysplasia stage (33% of dysplasia cases).^41^ In our study, *TFPI2* was the candidate with the best sensitivity in both the pilot and validation Cytosponge cohorts (85.0% and 78.6%, respectively, AUC 87.7%). This observation is consistent with Alvi’s methylome dataset in Barrett’s biopsies, although the number of samples was smaller^28^ (online supplementary figure 1B).

Similarly, TWIST1 is also known to be hypermethylated in various tumour types including bladder and colorectal cancers.^42 43^ We found that although TWIST1 was significantly hypermethylated in Barrett’s samples from both pilot and validation cohorts, the AUC was lower compared with TFPI2 (AUC=81.4% for TWIST1).

ZNF345 and ZNF569 are novel methylation markers that have not previously been reported to be hypermethylated in cancer. ZNF345 is the most specific biomarker with a specificity of 100% in both pilot and validation Cytosponge cohorts. This is especially important when considering a screening test for a disease with a low population prevalence estimated at approximately 3% in patients with reflux symptoms.^14^

It is possible that a combination of the biomarkers would outperform the individual biomarker analysis. However, this would require validation in an independent cohort of patients to avoid overfitting the data.

The demographics of our cohort was highly consistent with the literature in that Barrett’s cases were more likely to be: older, male, with an increased waist:hip ratios and a hiatus hernia.^7 44^ It is well known in the literature that increasing age is associated with changes in gene methylation status^21^; however, there was no significant difference in the age of our methylation signature-positive cases and methylation negative cases, confirming that age is not a confounding factor. Interestingly, cases that were negative for the methylation biomarkers had smaller circumferential (C) and maximal (M) lengths of Barrett’s (p<0.001 for both C and M). This is in keeping with previous studies that have reported a significant correlation between BE segment length and hypermethylation of other markers, such as AKAP12 and CHD13, for example.^22 35^ Furthermore, the sensitivity of TFF3 is also affected by the length of the Barrett’s oesophagus in the same cohort such that the overall sensitivity (79.9%) increased to 87.2% for patients with >3 cm circumferential Barrett’s oesophagus.^17^ Similarly, the sensitivity of our methylation signature increased from 82.2% to 95% for segments >3 cm (online supplementary figure 3). This finding is clinically relevant as it has been well documented that long segment BE are more likely to progress to OAC.^45–52^ It is envisaged that risk stratification for malignant potential could be performed using additional biomarkers on the same Cytosponge sample following evaluation for the Barrett’s oesophagus specific biomarker.^15^

Strengths of this study include the large validation cohort, although it should be noted that this was a retrospective analysis and prospective validation is required. We have shown that TFPI2 was hypermethylated in Barrett’s samples. Although the focus of this study was non-dysplastic Barrett’s oesophagus, it is interesting to note that Kaz *et al* 33 have shown that *TFPI2* methylation persists in high-grade dysplasia and adenocarcinoma. Corroborating these findings, using methylation data previously produced from our laboratory,^28^ we observe that methylation levels in BE and OAC are comparable (online supplementary figure 4). This is important since one would not want to miss dysplasia in a screening setting, and this suggests that methylation of this gene is preserved in oesophageal carcinogenesis.

An ideal screening test should be easy to perform, acceptable to patients and cost-effective. Our studies so far suggest that the Cytosponge fulfils these criteria with an excellent safety profile in over 2000 patients, high acceptability levels and applicability to the primary care setting.^14 17^ TFF3 is highly specific (92%–94%) with encouraging sensitivity levels (79.9%) especially in longer segments or after a second Cytosponge test (87.2% and 89.7%, respectively).^17^ Methylation of TFPI2, TWIST1, ZNF345 or ZNF569 has a comparable performance with BEST2, although it should be noted that the assays were performed on a subcohort due to sample availability. MethyLight PCR is an assay that could be semiautomated and implemented in a diagnostic laboratory without paraffin-embedding reliance on a pathological analysis of the slide. It should be noted that any diagnostic biomarker requires testing in the primary care cohort for which it is intended, and further prospective trials are warranted to further test the accuracy and clinical applicability of these methylation biomarkers.

## References

[1] Medical Research Council Oesophageal Cancer Working Group. Surgical resection with or without preoperative chemotherapy in oesophageal cancer: a randomised controlled trial. Lancet 2002;359:1727–33. 10.1016/S0140-6736(02)08651-8 12049861

[2] Lao-Sirieix P, Fitzgerald RC Screening for oesophageal cancer. Nat Rev Clin Oncol 2012;9:278–87. 10.1038/nrclinonc.2012.35 22430857

[3] Lepage C, Rachet B, Jooste V, et al Continuing rapid increase in esophageal adenocarcinoma in England and wales. Am J Gastroenterol 2008;103:2694–9. 10.1111/j.1572-0241.2008.02191.x 18853967

[4] Pohl H, Sirovich B, Welch HG Esophageal adenocarcinoma incidence: are we reaching the peak? Cancer Epidemiol Biomarkers Prev 2010;19:1468–70. 10.1158/1055-9965.EPI-10-0012 20501776

[5] Steevens J, Botterweck AA, Dirx MJ, et al Trends in incidence of oesophageal and stomach cancer subtypes in Europe. Eur J Gastroenterol Hepatol 2010;22:669–78. 10.1097/MEG.0b013e32832ca091 19474750

[6] Bosetti C, Levi F, Ferlay J, et al Trends in oesophageal cancer incidence and mortality in Europe. Int J Cancer 2008;122:1118–29. 10.1002/ijc.23232 17990321

[7] Fitzgerald RC, di Pietro M, Ragunath K, et al British society of gastroenterology guidelines on the diagnosis and management of Barrett’s oesophagus. Gut 2014;63:7–42. 10.1136/gutjnl-2013-305372 24165758

[8] Spechler SJ, Souza RF Barrett’s esophagus. N Engl J Med 2014;371:836–45. 10.1056/NEJMra1314704 25162890

[9] Vaughan TL, Fitzgerald RC Precision prevention of oesophageal adenocarcinoma. Nat Rev Gastroenterol Hepatol 2015;12:243–8. 10.1038/nrgastro.2015.24 25666644 PMC4382373

[10] Hvid-Jensen F, Pedersen L, Drewes AM, et al Incidence of adenocarcinoma among patients with Barrett’s esophagus. N Engl J Med 2011;365:1375–83. 10.1056/NEJMoa1103042 21995385

[11] Lochhead P, Chan AT Screening and surveillance for Barrett esophagus. JAMA Intern Med 2015;175:159–60. 10.1001/jamainternmed.2014.6983 25546002 PMC4331337

[12] Jobe BA, Hunter JG, Chang EY, et al Office-based unsedated small-caliber endoscopy is equivalent to conventional sedated endoscopy in screening and surveillance for Barrett’s esophagus: a randomized and blinded comparison. Am J Gastroenterol 2006;101:2693–703. 10.1111/j.1572-0241.2006.00890.x 17227516

[13] Ramirez FC, Akins R, Shaukat M Screening of Barrett’s esophagus with string-capsule endoscopy: a prospective blinded study of 100 consecutive patients using histology as the criterion standard. Gastrointest Endosc 2008;68:25–31. 10.1016/j.gie.2007.10.040 18499107

[14] Kadri SR, Lao-Sirieix P, O’Donovan M, et al Acceptability and accuracy of a non-endoscopic screening test for Barrett’s oesophagus in primary care: cohort study. BMJ 2010;341:c4372 10.1136/bmj.c4372 20833740 PMC2938899

[15] Ross-Innes CS, Chettouh H, Achilleos A, et al Risk stratification of Barrett’s oesophagus using a non-endoscopic sampling method coupled with a biomarker panel: a cohort study. Lancet Gastroenterol Hepatol 2017;2:23–31. 10.1016/S2468-1253(16)30118-2 28404010

[16] Benaglia T, Sharples LD, Fitzgerald RC, et al Health benefits and cost effectiveness of endoscopic and nonendoscopic cytosponge screening for Barrett’s esophagus. Gastroenterology 2013;144:62–73. 10.1053/j.gastro.2012.09.060 23041329

[17] Ross-Innes CS, Debiram-Beecham I, O’Donovan M, et al Evaluation of a minimally invasive cell sampling device coupled with assessment of trefoil factor 3 expression for diagnosing Barrett’s esophagus: a multi-center case-control study. PLoS Med 2015;12:e1001780 10.1371/journal.pmed.1001780 25634542 PMC4310596

[18] Weller D, Coleman D, Robertson R, et al The UK colorectal cancer screening pilot: results of the second round of screening in England. Br J Cancer 2007;97:1601–5. 10.1038/sj.bjc.6604089 18026197 PMC2360273

[19] Mistry K, Cable G Meta-analysis of prostate-specific antigen and digital rectal examination as screening tests for prostate carcinoma. J Am Board Fam Pract 2003;16:95–101. 10.3122/jabfm.16.2.95 12665174

[20] Ferrini R, Mannino E, Ramsdell E, et al Screening mammography for breast cancer: American college of preventive medicine practice policy statement. Am J Prev Med 1996;12:340–1. 8909643

[21] Laird PW The power and the promise of DNA methylation markers. Nat Rev Cancer 2003;3:253–66. 10.1038/nrc1045 12671664

[22] Jin Z, Hamilton JP, Yang J, et al Hypermethylation of the AKAP12 promoter is a biomarker of Barrett’s-associated esophageal neoplastic progression. Cancer Epidemiol Biomarkers Prev 2008;17:111–7. 10.1158/1055-9965.EPI-07-0407 18199717

[23] Wang JS, Guo M, Montgomery EA, et al DNA promoter hypermethylation of p16 and APC predicts neoplastic progression in Barrett’s esophagus. Am J Gastroenterol 2009;104:2153–60. 10.1038/ajg.2009.300 19584833 PMC3090447

[24] Eads CA, Lord RV, Wickramasinghe K, et al Epigenetic patterns in the progression of esophageal adenocarcinoma. Cancer Res 2001;61:3410–8. 11309301

[25] Peng DF, Razvi M, Chen H, et al DNA hypermethylation regulates the expression of members of the Mu-class glutathione S-transferases and glutathione peroxidases in Barrett’s adenocarcinoma. Gut 2009;58:5–15. 10.1136/gut.2007.146290 18664505 PMC2845391

[26] Lee OJ, Schneider-Stock R, McChesney PA, et al Hypermethylation and loss of expression of glutathione peroxidase-3 in Barrett’s tumorigenesis. Neoplasia 2005;7:854–61. 10.1593/neo.05328 16229808 PMC1501938

[27] Agarwal A, Polineni R, Hussein Z, et al Role of epigenetic alterations in the pathogenesis of Barrett’s esophagus and esophageal adenocarcinoma. Int J Clin Exp Pathol 2012;5:382–96. 22808291 PMC3396065

[28] Alvi MA, Liu X, O’Donovan M, et al DNA methylation as an adjunct to histopathology to detect prevalent, inconspicuous dysplasia and early-stage neoplasia in Barrett’s esophagus. Clin Cancer Res 2013;19:878–88. 10.1158/1078-0432.CCR-12-2880 23243219 PMC4998953

[29] Eads CA, Danenberg KD, Kawakami K, et al MethyLight: a high-throughput assay to measure DNA methylation. Nucleic Acids Res 2000;28:32e 10.1093/nar/28.8.e32 PMC102836 10734209

[30] Zou H, Osborn NK, Harrington JJ, et al Frequent methylation of eyes absent 4 gene in Barrett’s esophagus and esophageal adenocarcinoma. Cancer Epidemiol Biomarkers Prev 2005;14:830–4. 10.1158/1055-9965.EPI-04-0506 15824152

[31] Smith E, De Young NJ, Pavey SJ, et al Similarity of aberrant DNA methylation in Barrett’s esophagus and esophageal adenocarcinoma. Mol Cancer 2008;7:75 10.1186/1476-4598-7-75 18831746 PMC2567345

[32] Zou H, Molina JR, Harrington JJ, et al Aberrant methylation of secreted frizzled-related protein genes in esophageal adenocarcinoma and Barrett’s esophagus. Int J Cancer 2005;116:584–91. 10.1002/ijc.21045 15825175

[33] Kaz AM, Wong CJ, Luo Y, et al DNA methylation profiling in Barrett’s esophagus and esophageal adenocarcinoma reveals unique methylation signatures and molecular subclasses. Epigenetics 2011;6:1403–12. 10.4161/epi.6.12.18199 22139570 PMC3256330

[34] Clément G, Braunschweig R, Pasquier N, et al Methylation of APC, TIMP3, and TERT: a new predictive marker to distinguish Barrett’s oesophagus patients at risk for malignant transformation. J Pathol 2006;208:100–7. 10.1002/path.1884 16278815

[35] Jin Z, Cheng Y, Olaru A, et al Promoter hypermethylation of CDH13 is a common, early event in human esophageal adenocarcinogenesis and correlates with clinical risk factors. Int J Cancer 2008;123:2331–6. 10.1002/ijc.23804 18729198

[36] Sun FK, Sun Q, Fan YC, et al Methylation of tissue factor pathway inhibitor 2 as a prognostic biomarker for hepatocellular carcinoma after hepatectomy. J Gastroenterol Hepatol 2016;31:484–92. 10.1111/jgh.13154 26313014

[37] Park SK, Song CS, Yang HJ, et al Field cancerization in sporadic colon cancer. Gut Liver 2016;10:773–80. 10.5009/gnl15334 27114416 PMC5003201

[38] Liu Z, Zhang J, Gao Y, et al Large-scale characterization of DNA methylation changes in human gastric carcinomas with and without metastasis. Clin Cancer Res 2014;20:4598–612. 10.1158/1078-0432.CCR-13-3380 25009298 PMC4309661

[39] Li YF, Hsiao YH, Lai YH, et al DNA methylation profiles and biomarkers of oral squamous cell carcinoma. Epigenetics 2015;10:229–36. 10.1080/15592294.2015.1006506 25612142 PMC4622594

[40] Glöckner SC, Dhir M, Yi JM, et al Methylation of TFPI2 in stool DNA: a potential novel biomarker for the detection of colorectal cancer. Cancer Res 2009;69:4691–9. 10.1158/0008-5472.CAN-08-0142 19435926 PMC3062162

[41] Jia Y, Yang Y, Brock MV, et al Methylation of TFPI-2 is an early event of esophageal carcinogenesis. Epigenomics 2012;4:135–46. 10.2217/epi.12.11 22449186 PMC3742137

[42] van Kessel KE, Van Neste L, Lurkin I, et al Evaluation of an epigenetic profile for the detection of bladder cancer in patients with hematuria. J Urol 2016;195:601–7. 10.1016/j.juro.2015.08.085 26327355

[43] Lin PC, Lin JK, Lin CH, et al Clinical relevance of plasma DNA methylation in colorectal cancer patients identified by using a genome-wide high-resolution array. Ann Surg Oncol 2015;22(suppl 3):1419–27. 10.1245/s10434-014-4277-2 25472652

[44] de Jonge PJ, van Blankenstein M, Grady WM, et al Barrett’s oesophagus: epidemiology, cancer risk and implications for management. Gut 2014;63:191–202. 10.1136/gutjnl-2013-305490 24092861 PMC6597262

[45] Wani S, Falk GW, Post J, et al Risk factors for progression of low-grade dysplasia in patients with Barrett’s esophagus. Gastroenterology 2011;141:1179–86. 10.1053/j.gastro.2011.06.055 21723218

[46] Sikkema M, Looman CW, Steyerberg EW, et al Predictors for neoplastic progression in patients with Barrett’s esophagus: a prospective cohort study. Am J Gastroenterol 2011;106:1231–8. 10.1038/ajg.2011.153 21577245

[47] Weston AP, Sharma P, Mathur S, et al Risk stratification of Barrett’s esophagus: updated prospective multivariate analysis. Am J Gastroenterol 2004;99:1657–66. 10.1111/j.1572-0241.2004.30426.x 15330898

[48] Hage M, Siersema PD, van Dekken H, et al Oesophageal cancer incidence and mortality in patients with long-segment Barrett’s oesophagus after a mean follow-up of 12.7 years. Scand J Gastroenterol 2004;39:1175–9. 10.1080/00365520410003524 15742992

[49] Rudolph RE, Vaughan TL, Storer BE, et al Effect of segment length on risk for neoplastic progression in patients with Barrett esophagus. Ann Intern Med 2000;132:612–20. 10.7326/0003-4819-132-8-200004180-00003 10766679

[50] Hirota WK, Loughney TM, Lazas DJ, et al Specialized intestinal metaplasia, dysplasia, and cancer of the esophagus and esophagogastric junction: prevalence and clinical data. Gastroenterology 1999;116:277–85. 10.1016/S0016-5085(99)70123-X 9922307

[51] O’Connor JB, Falk GW, Richter JE The incidence of adenocarcinoma and dysplasia in barrett’s esophagus: report on the cleveland clinic Barrett’s esophagus registry. Am J Gastroenterol 1999;94:2037–42. 10.1111/j.1572-0241.1999.01275.x 10445525

[52] Menke-Pluymers MB, Hop WC, Dees J, et al Risk factors for the development of an adenocarcinoma in columnar-lined (Barrett) esophagus. The rotterdam esophageal tumor study group. Cancer 1993;72:1155–8. 8339208 10.1002/1097-0142(19930815)72:4<1155::aid-cncr2820720404>3.0.co;2-c

