# Supplementary material for: Methylation panel is a diagnostic biomarker for Barrett’s oesophagus in endoscopic biopsies and non-endoscopic cytology specimens
Source: Gut. 2017 Oct 30;67(11):1942–9. doi: 10.1136/gutjnl-2017-314026 (PMC6176521; doi:10.1136/gutjnl-2017-314026)
Supplement: Supplementary file 2 [file gutjnl-2017-314026supp002.pdf]

Sequence Definition	Probe Sequence	Sense Primer	Anti-sense Primer
TFPI2	[6FAM]CGTTTGGCGGGAGGAGGTGCGCGGTT[TAM]	TGTAGGGGGTCGGGCGGTTC	CGCTCGCCCCGCATAAAACG
ALDH1A3_1	[6FAM]CCCTACGACTTCCGAAACCCGACGA[TAM]	AACTCCCTACCGAATCGAATATCC	GTTCGTTTTATTTCGTTTCGTTCGT
ALDH1A3_2	[6FAM]AAACCCGCCTACAACGTAAACGCAAATCT[TAM]	TTTATCCCCGAATCGACACGAAAA	ATTAGGTTGTTGAGGTTAATATAGAAACG
APC	[6FAM]CAACAACACCTCTCACGCATACGCATTAT[TAM]	GCATAACAAACTCTAATCTCCGAAC	TAGGTTTTCGGCGGTTATATTTCG
CCND2_1	[6FAM]CTCACGCCTATCGCATCTACCGCCT[TAM]	ATAAATAACAAACGACAAACGCTCG	AGAGTTCGTTAGGTTTGTGTATTCG
CCND2_2	[6FAM]AACGCTACCTTCCGCAATACTCCTACTTC[TAM]	CTACAAAACCTACTCACCATCGAA	ATTATTTTGCGTATGTAGGGTTGGA
CDKN2B	[6FAM]CGACCGCTCAAACGCTCCAAATCCAC[TAM]	AATCCACTACCCGCCTAACTACTC	CGTTTGGTATTGTGAGTAATTATTGAGAT
ELMO1	[6FAM]TATACCCATCCTCGCCCGCTCCCG[TAM]	CGACGACGTAAATAACTCTACCTC	GGTGTAGTAGTCGGTCGATTTAGA
EYA4	[6FAM]ACGCCTAAACTTTCCCGATACCGAATACG[TAM]	GAAAATACTAACCCAAAATCCTTCGA	CGATTAGTGTTTGGAGATTAGAACG
HOXD4_1	[6FAM]ACGATCCCGAACGACCTACACCCG[TAM]	CAATAAACCCCAACTACACCGATA	ATTTTAATTCGACGGCGTTTTGTTA
HOXD4_2	[6FAM]CGCCGACTACTACGACGACGACGC[TAM]	AAATATTTACAAAACGACTACCTAAACGAA	TTGTTCGTGATTTCGCGTAGGTAG
NEUROG1	[6FAM]CCTTAAATACCCGACGCAACAATAAACGCC[TAM]	GAATATAACACACGACTAACCTCAAAA	TGATTTGTCGTTTCGTTTTATTTATTCG
PTPRO	[6FAM]CGCCATTATAAACCGCCGCCGAAA[TAM]	GACGCCCAAAATCTAAAACTACAAC	TTTTCGTGGGTAGGTGTTTTATCG
RBP1	[6FAM]ACCCGAAAATAAACGCCCTCCGAAAACA[TAM]	CTACCGATACTACGCGAATAATAAAC	GAATTTAGTTGTCGTCGTTTCGTA
RPIB9	[6FAM]TATTCCCAAACCCGAACTCCCGATTCCG[TAM]	GAAACCGCGTCGTTCCTAAACTTA	GTTTAAGGAGGAGCGTGGTGTTAG
SFRP4	[6FAM]AACAAACAACTCGTCGCAAACCAAACTTTC[TAM]	ATAACTTCAAACGAAATACACACGC	TTTATTAAGTCGTGTAAGTCGGTGT
SFRP4	[6FAM]TTACCAAATCTCGTCGCAACCGCCG[TAM]	CTACTCCTAAAACCGACGTTTAACT	GGGTATTTTGAGGTTGTGTATTCG
TWIST1	[6FAM]ACTCCCGCCGCCGCTACTACTACC[TAM]	CGTCTACAACTCCTCGTAAAACTACG	TCGGGTAGTTCGGTTTAGGGTAAG
ZNF345_1	[6FAM]ACGTAAATAAAACGACTCGAAACCTAACGC[TAM]	TAAATTCTAATAAAACGAAACTAACCCTAT	TACGTAGTTTATATAGGTCGGGAG
ZNF345_2	[6FAM]CGACTCCTCCACACGCTACGCAACTAC[TAM]	ACGCTCAAATACCACATTAAAATACG	TTAATTGTGTTTCGGAGGTTGTTTG
ZNF569_1	[6FAM]CGCCACAAATCTCGATCCGTTACACCAA[TAM]	GACACTAAAACCGACGCTATCGAT	GAAGTTAGTTTCGTTCGGGTGAGT
ZNF569_2	[6FAM]CCCGTCCCTATAACCGAACCAAACTCG[TAM]	CCACACCAACCCGTATAATATAACG	GTAGTTTCGCGTAGGGTTTGTAAT
ZNF625	[6FAM]TCCATAACCCTCCCGTAATCTCCGCAC[TAM]	ACCACACACCCGAAACAAAATTAC	GTAGTCGGGATTTTAGGCGTTTTG
beta-actin	[6FAM]ACCACCACCCAACACACAATAACAAACACA[TAM]	TGGTGATGGAGGAGGTTTAGTAAGT	AACCAATAAAACCTACTCCTCCCTTAA
