# Supplementary material for: Methylation panel is a diagnostic biomarker for Barrett’s oesophagus in endoscopic biopsies and non-endoscopic cytology specimens
Source: Gut. 2017 Oct 30;67(11):1942–9. doi: 10.1136/gutjnl-2017-314026 (PMC6176521; doi:10.1136/gutjnl-2017-314026)
Supplement: Supplementary file 1 [file gutjnl-2017-314026supp001.pdf]

Supplementary Figure 1: Barrett's Methylation candidates

These data were from Alvi et al 2013. (A) Boxplots representing the methylation level of the differentially methylated genes in Barrett's compared to normal tissue. (B) Heatmap representing the non-supervised hierarchical clustering of patients according to the methylation level of genes of interest.

Supplementary Figure 2: Methylation level of B3GAT2, CDX2 and Vimentin

Boxplots representing the methylation level of the B3GAT2, CDX2 and Vimentin (VIM) genes in Barrett's compared to normal tissue from Alvi et al 2013 dataset.

Supplementary Figure 3: Methylation levels of our identified markers according to the length of the Barrett's segment using the Cytosponge™.

Dotplot presenting sensitivity variation by circumferential (C) and maximal (M) Barrett's length.

Supplementary Figure 4: Methylation status in dysplasia and adenocarcinoma.

Boxplot of normalised beta values showing hypermethylation of each gene candidate in Barrett's and Oesophageal adenocarcinoma biopsies compared to normal tissue – data extracted from Alvi's dataset (Alvi et al., 2013)

References

Alvi MA, Liu X, O'Donovan M, et al. DNA methylation as an adjunct to histopathology to detect prevalent, inconspicuous dysplasia and early-stage neoplasia in Barrett's esophagus. *Clin Cancer Res* 2013;19(4):878-88. doi: 10.1158/1078-0432.CCR-12-2880

Confidential: For Review Only

Supplementary Figure 1

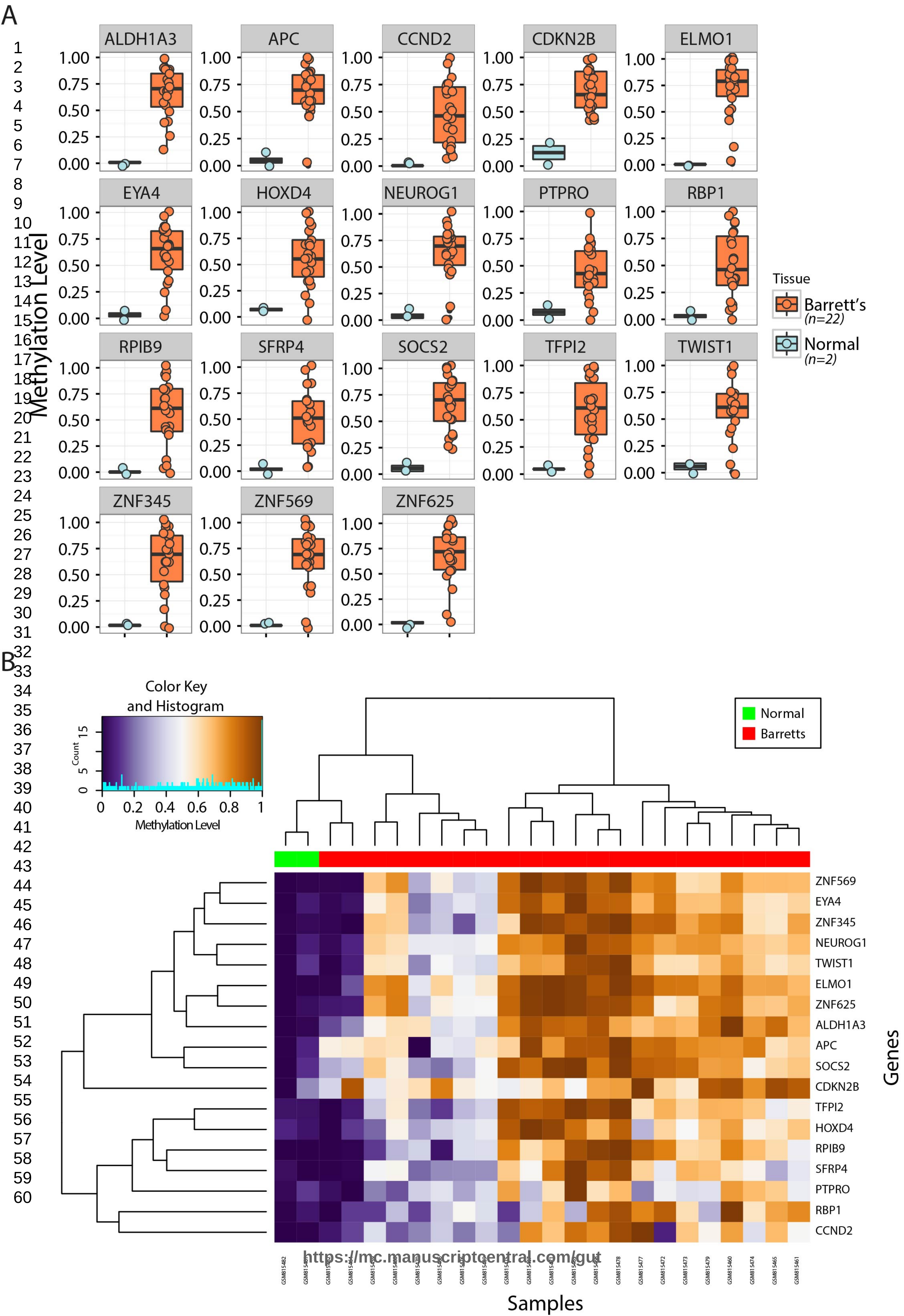

https://mc.manuscriptcentral.com/gut

Supplementary Figure 2

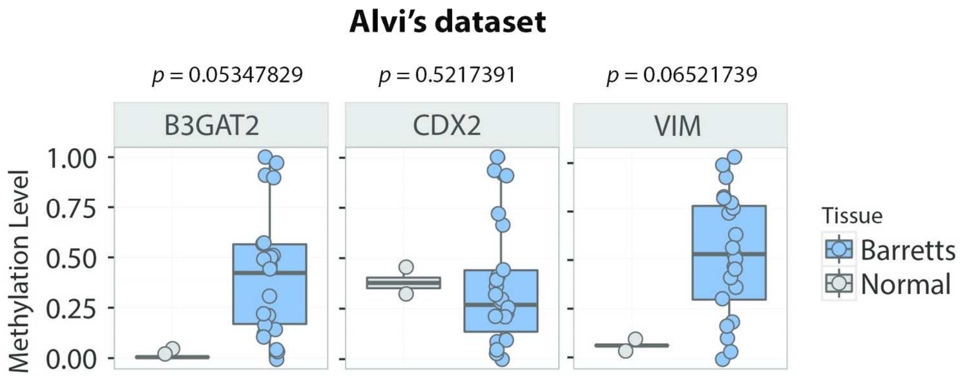

178x78mm (300 x 300 DPI)

For Review Only

Supplementary Figure 3

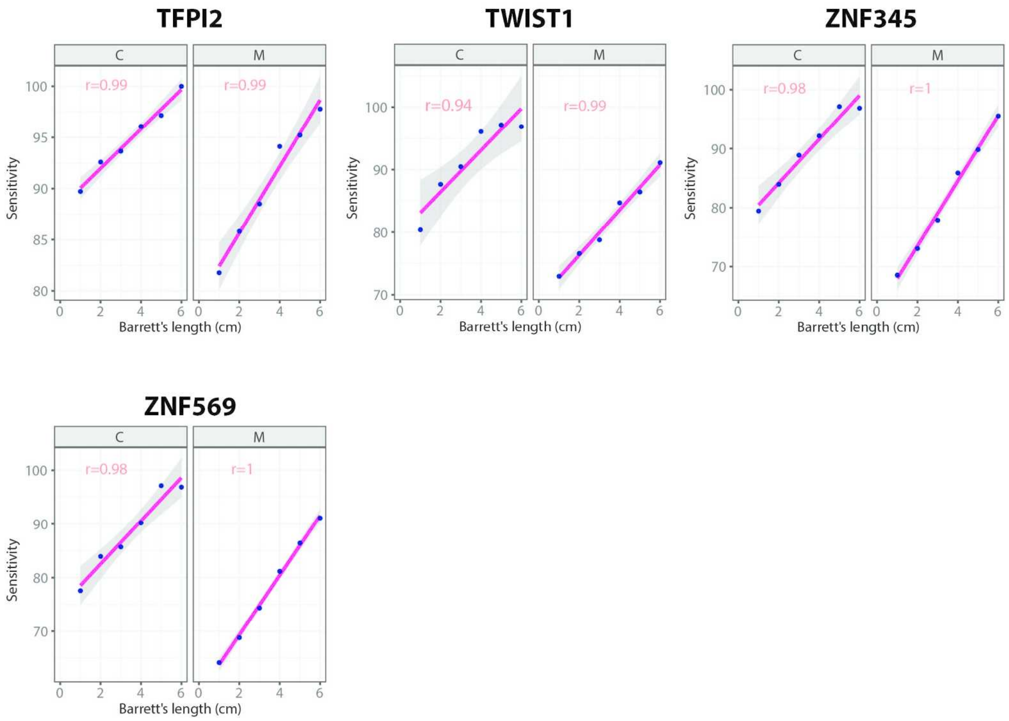

198x156mm (300 x 300 DPI)

Review Only

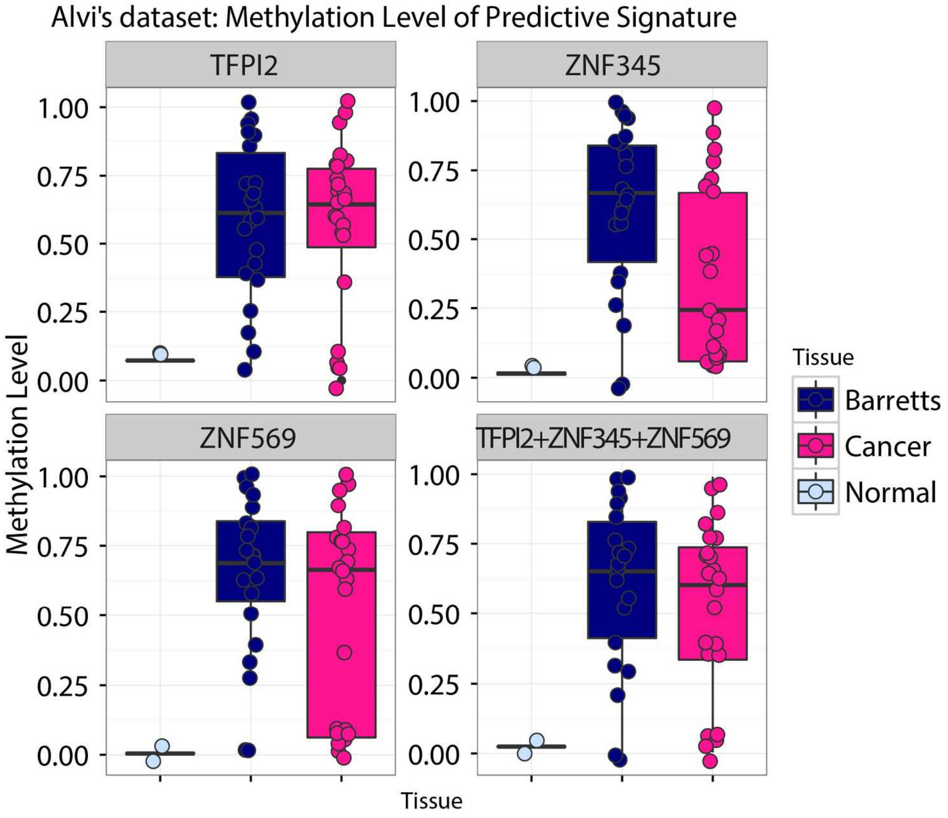

176x164mm (300 x 300 DPI)

iew Only
